# Systematic Review of Avian Influenza Virus Infection and Outcomes during Pregnancy

**DOI:** 10.3201/eid3101.241343

**Published:** 2025-01

**Authors:** Rachael Purcell, Michelle L. Giles, Nigel W. Crawford, Jim Buttery

**Affiliations:** Monash Health, Clayton, Victoria, Australia (R. Purcell, M.L. Giles); University of Melbourne, Melbourne, Victoria, Australia (R. Purcell, M.L. Giles, N.W. Crawford, J. Buttery); Murdoch Children’s Research Institute, Melbourne (R. Purcell, N.W. Crawford, J. Buttery); Royal Children’s Hospital, Melbourne (R. Purcell, N.W. Crawford, J. Buttery); Monash University, Clayton (M.L. Giles); Global Vaccine Data Network, Auckland, New Zealand (J. Buttery)

**Keywords:** avian influenza virus, influenza, viruses, zoonoses, respiratory infections, pregnancy, infection, vaccination, pandemic, Australia

## Abstract

Human cases of avian influenza A(H5N2) and A(H5N1) viruses associated with outbreaks in birds and mammals are increasing globally, raising concerns about the possibility of a future avian influenza pandemic. We conducted a systematic review examining 30 reported cases of avian influenza in pregnant women. We found high mortality rates for mothers (90.0%, 27/30) and their babies (86.7%, 26/30) when women were infected with avian influenza virus during pregnancy. Despite being a high-risk population and having worse health outcomes across multiple pandemics, pregnant women are often excluded from vaccine trials. However, as the risk for a new pandemic increases and human vaccines against avian influenza are developed, early inclusion of pregnant women in clinical trials can inform the risk–benefit analysis for both the mother and their newborn infant. Early inclusion of pregnant women in public health vaccination programs is vital for protecting this high-risk population.

During pandemics, special risk populations are often more vulnerable to severe disease and death. Pregnant women experienced higher mortality and critical illness rates during the 2009 influenza pandemic (*1*), the SARS-CoV-2 pandemic (*2*), and the 2014–2016 Ebola epidemic in Africa (*3*). Global efforts are needed to proactively recognize and mitigate risks to pregnant women before the onset of a pandemic, rather than as a reactive process after a pandemic has started.

Recent case reports of human infection with avian influenza A(H5N2) and A(H5N1) viruses have renewed concerns regarding the heightened risk for a pandemic. An increasing number of cases of human avian influenza virus infection have been reported since 2004, and increasing case numbers have been seen since 2023 (*4*), exceeding 1,400 cases across different subtypes (*5*). Avian influenza is commonly caused by influenza A virus subtypes H5, H7, and H9. Occurring naturally among wild water birds, a rising number of avian influenza infections have been reported in domestic poultry and in mammals, including domestic cats and dogs and humans (*6*).

In April 2024, a human case of H5N2 virus infection was described in a 59-year-old man from Mexico who later died (*7*). The man had no known contact with infected animals, but cases of infected poultry were detected in the neighboring Mexican state in March 2024 (*7*). That case was the first known human case of A(H5N2) influenza virus infection, although seropositivity has been previously described in poultry workers in Japan after a large avian outbreak in 2005 (*8*). The possibility that seasonal influenza vaccination influences H5N2 virus neutralizing antibody titers makes understanding seroepidemiology and risk for human infection challenging (*9*).

Of similar concern are increasing cases of influenza A(H5N1) virus infection in animals. Sporadic cases of infections in mammals have been described in Europe, South America, North America, and Asia (*6*). Unexplained illness in dairy cattle leading to decreased milk production was described in the United States in January 2024 (*10*). Influenza A(H5N1) virus was later detected in cattle in March 2024, as was a human case of infection after exposure to dairy cows in April 2024 (*11*). Illness in other animals has also occurred, including detection in foxes, sea elephants, and sea lions, as well as in domestic animals, such as dogs and cats (*6*). Symptomatic human infection has also occurred in Cambodia, where 5 human cases related to infected poultry were reported in early 2024 (*12*). Other case reports have emerged, including a case in Vietnam after exposure to wild birds, a child in Australia who had recently traveled to India (*13*), and 17 cases in the United States, including 16 patients who had contact with infected dairy cows or poultry (*14*).

In previous influenza pandemics, pregnant women experienced worse health outcomes and higher mortality rates than the general population. In some studies, pregnant women accounted for up to 9% of intensive care unit (ICU) admissions and up to 10% of patients who died (*1*). The risk for severe disease or adverse outcomes among pregnant women was observed again during the COVID-19 pandemic (*2*), before the introduction of vaccination, when pregnant women were at an increased risk for critical illness requiring ICU admission, extracorporeal membrane oxygenation, or mechanical oxygenation compared with nonpregnant women of a similar age.

Despite the increased risks, in the past, pregnant women have been excluded from clinical prelicensure trials of vaccines and therapeutic agents aiming to address pandemics (*15*,*16*). Pregnant women also have been excluded or have had delayed entry into population-level public health vaccination programs (*15*). As avian influenza virus infections in humans increase (*11*,*13*,*17*), understanding which populations are likely to be most vulnerable will be critical to pandemic preparedness efforts. We conducted a systematic review of avian influenza virus infection during pregnancy to assess adverse effects among this population.

## Methods

### Search Strategy

We followed Preferred Reporting Items for Systematic Reviews and Meta-Analyses (PRISMA) guidelines to conduct a systematic review of avian influenza virus during pregnancy and its effects on pregnancy outcomes. We searched MEDLINE (https://www.nlm.nih.gov/medline/medline_home.html) and EMBASE (https://www.embase.com) databases from inception through June 2024 for original studies. We identified additional records through reference checking. The studies included pregnant women who had experienced an avian influenza virus infection during any stage of pregnancy. We included studies that reported on pregnancy outcomes.

### Study Selection Process and Data Extraction

We searched databases and reviewed titles and abstracts for each study, then we removed duplicate studies from search results. Two independent reviewers screened all abstracts and full texts selected for retrieval. The authors reviewed full text for articles that met the study inclusion criteria. We extracted and compiled data in a PRISMA format table, including study design, setting, number of participants, intervention group or population, and outcomes (Table).

**Table:** Summary of included studies*

Reference	Study design	Country of origin	Sample size	Virus strain	Diagnostic method	Population	Outcomes
(*18*)	RSCS	China, Egypt, Indonesia	23	H7N9	PCR	10 pregnant women, mean age 28 y (range 20–35 y); GA at time of infection: trimester 1 (n = 3), trimester 2 (n = 4), trimester 3 (n = 3)	9 maternal and 8 in utero fetal deaths. One infant born prematurely at 33 weeks GA to a mother who later died. The woman who survived was at 9-weeks GA at time of infection and her infant survived.
				H5N1	PCR	13 pregnant women, mean age 24.8 y (range 20–35); GA at time of infection: trimester 1 (n = 1), trimester 2 (n = 7), trimester 3 (n = 2), unknown GA (n = 5)	13 maternal and 12 in utero fetal deaths. Delivery of 1 infant via emergency caesarean section at 36 weeks GA before maternal death; infant was LBW (2.3 kg), was not infected, and survived.
(*19*)	Case report	Vietnam	1	A(H5N1) clade 1.1	PCR and genome sequencing of tracheal aspirate (maternal); throat swabs, serum (neonate)	26 years of age, 36 weeks GA; worked slaughtering poultry	Maternal death, newborn survived. Premature birth with LBW and early onset pneumonia. Infant recovered by day 16 of life. PCR and serum specimens for H5N1 negative.
(*20*)	Case report	China	1	A(H5N1)	PCR and genome sequencing of tracheal aspirate	Age unknown, 16 weeks GA, who slaughtered sick poultry	Maternal and in utero fetal death
(*21*)	Case report	China	1	A(H5N1)	PCR	Age and gestation unknown, had contact with poultry.	Maternal and in utero fetal death
(*22*)	Case report	China	1	A(H5N6)	PCR of throat swab, sputum	40 years of age, 35 weeks GA; contact with poultry unknown	Maternal survival; live birth of premature infant, 35 weeks GA. No infant infection
(*23*)	Case report	China	1	A(H7N9)	PCR	29 years of age, 27 weeks GA; contact with poultry unknown	Maternal and in utero fetal death
(*24*)	Case report	China	1	A(H7N9)	PCR of tracheal aspirate	28 years of age, 26 weeks GA; contact with poultry unknown	Maternal and in utero fetal death
(*25*)	Case report	China	1	A(H7N9)	Unknown	25 years of age, 17 weeks GA; visited live animal market 2 weeks before illness	Maternal survival; infant born at 35 weeks GA, 2 mo after maternal hospital discharge

*All the adult population described in the table are pregnant women. GA, gestational age; LBW, low birthweight; RSCS, retrospective cohort study.

### Inclusion and Exclusion Criteria

Studies included for full text review were randomized or nonrandomized controlled trial studies, cohort studies, retrospective or prospective observational studies, or case series or case reports. Because many included articles were from China, we included studies published in Mandarin and had those translated by a local author. We excluded studies that did not report on primary outcome, those in which pregnant women were not differentiated from other study participants, and those reporting duplicate data.

### Definitions Related to Outcomes of Interest

We defined preterm birth as any live birth before 37 completed gestational weeks. We only included avian influenza virus infections in humans for analysis.

## Results

### Study Selection

After removal of duplicate studies, we identified a total of 1,602 publications (Figure). From those, we excluded 1,592 studies after abstract screening and conducted a full text review for 10 studies, 8 of which we included for analysis. Reasons for exclusion included that the population described was not pregnant women, the pregnancy outcome was not known, or the case report was a duplication of a previously described patient (*26*) (Figure). Included studies were 7 individual case reports and 1 retrospective cohort study. To avoid duplication, we removed 3 persons described in individual case reports (*20*,*25*,*27*) from our description of the retrospective cohort because those persons were also described in the retrospective cohort study (*18*).

**Figure:**
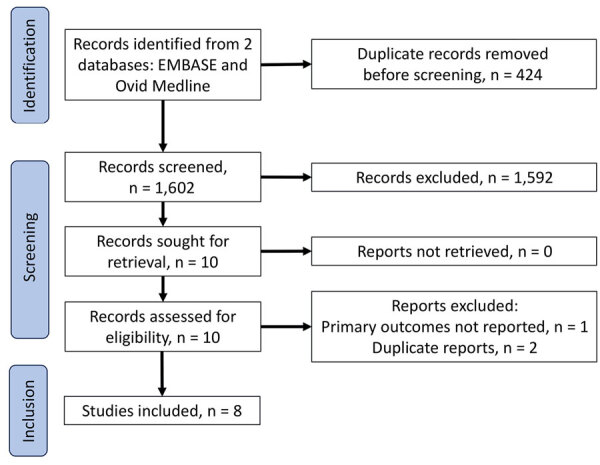
Flow diagram for study review and inclusion in a systematic review of avian influenza virus infection and outcomes during pregnancy.

The review included a population of 30 pregnant women with diagnosed avian influenza virus infection during pregnancy (Table). The women resided across 4 countries, and most were from China. One review reported on patients from multiple countries (*18*). The avian influenza virus strains described included H5N1 (n = 16), H7N9 (n = 13), and H5N6 (n = 1). Microbiological diagnosis was made via PCR of tracheal aspirates, throat swab samples, or sputum, followed by genomic sequencing. Serologic testing in conjunction with PCR testing was used to exclude infection in 1 surviving neonate (*19*).

Exposure to poultry, either through attendance at live poultry markets or working with live poultry (n = 12), or contact with sick poultry (n = 15) was common among included cases. The described incubation period across the cases was 1–10 days. The maternal age range was 20–35 years, and gestational age at the time of infection was 8–36 weeks. No study reported seasonal influenza vaccination status of affected women in the year of their infection.

Maternal and infant outcomes were poor. Maternal death occurred in 90.0% (n = 27) of cases. In most (86.6%, 26/30) cases, fetuses died with the mothers. Of the 5 infants who survived, 4 were born prematurely (range 33–36 weeks’ gestation). Three of those births occurred at the time of maternal infection, either by spontaneous labor or emergency caesarean section (*18*,*19*,*22*). One infant was born prematurely at 35 weeks’ gestation, 2 months after maternal infection (*25*). The timing of infection during pregnancy did not appear to influence the likelihood of maternal or infant survival.

## Discussion

Limited information on avian influenza virus infections in pregnancy is available, and we found only 30 reported cases despite >1,400 infected humans described in the literature (*5*). What we do know from the cases reported is that outcomes for mothers and their fetuses were poor, and most cases ended in both maternal and in utero fetal death. Although less severe cases could be more likely to be detected and reported, the limited description of outcomes for pregnant women infected with avian influenza virus paints a concerning picture.

We noted no obvious pattern between the timing of infection during pregnancy and maternal or fetal and infant outcomes. We found no reported cases of pregnant women infected with H5N2 virus. The last case of H5N1 virus infection in a pregnant woman was reported in 2019 (*23*), and we found no cases associated with the outbreaks occurring in 2024 through June.

Pregnancy is a unique physiologic state, and often renders otherwise healthy women vulnerable to worse outcomes after some infections than nonpregnant persons. That phenomenon has been seen in multiple infectious disease epidemics, including influenza (*1*), the COVID-19 pandemic (*25*), and viral hemorrhagic fever outbreaks, such as Lassa fever and Ebola (*3*,*26*). Health impacts of infections are not limited to the mother, and reports of premature birth or infant death related to maternal death are increasing. Adverse effects on the infant have been noted not only with maternal death, but also among infants born to women admitted to an ICU (*27*).

The viral infections described in poultry and other mammals in 2024 are primarily avian viruses, and no genetic changes which would increase transmission to, and between, humans have been observed (*28*). A 2024 report on H5N1 viral whole-genome sequencing from dairy cattle, birds, domestic cats, and a raccoon in association with epidemiologic data supported efficient cow-to-cow transmission, increasing concern about the potential ability of avian influenza H5N1 clade 2.3.4.4b to cross species and efficiently transmit within new species (*29*). On April 23, 2024, joint analysis of the World Health Organization, World Organisation for Animal Health, and Food and Agriculture Organization of the United Nations assessed the overall public health risk posed by H5N1 as low (*30*). However, persons who have exposure to infected animals or contaminated environments are at an increased risk level (*31*). Nonetheless, efforts in vaccine development have been made to provide human protection against acquisition and severe disease, and antigenic analysis demonstrated that the avian influenza virus detected in humans in 2024 would be well covered by candidate vaccine viruses (*31*). A vaccine for humans against H5N1 influenza virus has been developed, and major investments have been made to promote development using mRNA technology (*32*).

The European Commission’s Health Emergency Preparedness and Response Authority procured 665,000 zoonotic avian influenza virus vaccine doses (*33*), and vaccination for persons working in high-risk occupations, such as fur farmers, was commenced in Finland (*33*). However, vaccination during pregnancy is listed as a contraindication, and health providers cautioned against vaccinating pregnant women because of insufficient safety data (*34*).

The inclusion of women as early as possible is a key priority in pandemic planning (*16*,*35*). The presumption of inclusion described in advocacy attempts to change the default approach and aims to normalize the position of pregnant women being included in vaccine development, research, and deployment programs (*36*). Although efforts have been made by leading public health bodies to preempt the impact of respiratory viral pandemics on pregnant women (*37*), those efforts have yet to result in a universal systemic approach. During the COVID-19 pandemic, pregnant women were largely excluded from vaccine trials, and only 2 of 90 studies included pregnant women (*15*). Although the speed of SARS-CoV-2 vaccine development was unprecedented, the noninclusion of pregnant women, who were known to experience more severe infections than the general population (*2*), highlights how inclusion and equality of access to vaccination remains a core issue.

Ethical pandemic preparedness to avoid preventable deaths requires early inclusion of vulnerable populations in vaccine development, monitoring, and trials (*38*,*39*). The dogma of presumptive exclusion of pregnant women needs to change (*36*,*38*), and a pregnancy-focused research agenda should be developed and implemented by ethically informed oversight from institutional review boards, regulators, and policy makers (*38*).

Harnessing existing monitoring systems and resources to identify and include pregnant women and infant outcomes through use of administrative endpoints (*38*)—for example by using International Classification of Diseases, 10th Revision ,codes (*37*) —could be a method of providing systematic and timely prospective data. That strategy must be parallelled with a commitment to the rapid development and deployment of codes used to report new infections. Investment in the upkeep and readiness of pregnancy registries is also required. Although platforms in some jurisdictions have data available in a timely manner (*40*), others often report outcomes several years later (*39*). As recommended by the Pregnancy Research Ethics for Vaccines, Epidemics, and New Technologies (PREVENT) Working Group, suitability for administration during pregnancy should be a consideration when funding bodies are reviewing vaccine candidates, and the early initiation of preclinical development toxicology studies should be prioritized before efficacy studies (*38*). Vaccine trials that include women of childbearing age should be structured to systematically collect data on pregnancy-related safety outcomes and immunogenicity in the event of pregnancy occurring (*36*). Those data may also help inform outcomes from vaccine exposures earlier in pregnancy than would occur in planned antenatal vaccine trials. Similarly, using existing Rapid Cycle Analyses structures (*41*), such as the signal detection systems developed by the Global Vaccine Data Network or the Vaccine Safety Datalink (*40*,*42*), gives real-time data around population level vaccine safety.

## Conclusions

We used PRISMA guidelines to conduct a systematic review of avian influenza virus during pregnancy to assess infection effects on pregnancy outcomes. We found limited reports of outcomes for pregnant women infected with avian influenza virus in the literature. Of those reports, mortality rates for infected women and their infants was >90%.

As human cases of avian influenza A(H5N1) and A(H5N2) virus infection increase, awareness of the vulnerability of pregnant women to a new pandemic is needed. A paradigm shift is required to routinely include that population in pandemic preparedness programs and avoid preventable deaths. Inclusion could be achieved through using the capacity of existing surveillance systems, planning vaccine trials to include the complex needs of pregnancy, and scaling up signal detection systems to identify pregnancy outcomes.

## References

[1] Jamieson DJ, Honein MA, Rasmussen SA, Williams JL, Swerdlow DL, Biggerstaff MS, et al.; Novel Influenza A (H1N1) Pregnancy Working Group. H1N1 2009 influenza virus infection during pregnancy in the USA. Lancet. 2009;374:451–8. 10.1016/S0140-6736(09)61304-0 19643469

[2] Wei SQ, Bilodeau-Bertrand M, Liu S, Auger N. The impact of COVID-19 on pregnancy outcomes: a systematic review and meta-analysis. CMAJ. 2021;193:E540–8. 10.1503/cmaj.202604 33741725 PMC8084555

[3] Menéndez C, Lucas A, Munguambe K, Langer A. Ebola crisis: the unequal impact on women and children’s health. Lancet Glob Health. 2015;3:e130. 10.1016/S2214-109X(15)70009-4 25618242

[4] Centers for Disease Control and Prevention. Past reported global human cases with highly pathogenic avian influenza A(H5N1) (HPAI H5N1) by country, 1997–2024 [cited 2024 Dec 9]. https://www.cdc.gov/bird-flu/php/avian-flu-summary/chart-epi-curve-ah5n1.html

[5] Jernigan DB, Cox NJ. H7N9: preparing for the unexpected in influenza. Annu Rev Med. 2015;66:361–71. 10.1146/annurev-med-010714-112311 25386931

[6] Centers for Disease Control and Prevention. Highly pathogenic avian influenza A(H5N1) virus in animals: interim recommendations for prevention, monitoring, and public health investigations [cited 2024 Oct 5]. https://www.cdc.gov/bird-flu/prevention/hpai-interim-recommendations.html

[7] Mahase E. Bird flu: First person with confirmed H5N2 infection dies. BMJ. 2024;385:q1260. 10.1136/bmj.q1260 38849131

[8] Yamazaki Y, Doy M, Okabe N, Yasui Y, Nakashima K, Fujieda T, et al. Serological survey of avian H5N2-subtype influenza virus infections in human populations. Arch Virol. 2009;154:421–7. 10.1007/s00705-009-0319-7 19189196

[9] Joob B, Wiwanitkit V. Human H5N2 bird flu infection: fact or fallacy? Asian Pac J Trop Biomed. 2014;4(Suppl 1):S49. 10.12980/APJTB.4.2014C1202 25183136 PMC4025352

[10] US Department of Agriculture Animal and Plant Health Inspection Service. HPAI in livestock. 2024 [cited 2024 Dec 9]. https://www.aphis.usda.gov/livestock-poultry-disease/avian/avian-influenza/hpai-livestock

[11] Centers for Disease Control and Prevention. CDC A(H5N1) bird flu response update, July 19, 2024 [cited 2024 Aug 5]. https://www.cdc.gov/bird-flu/spotlights/h5n1-response-07192024.html

[12] World Health Organization. Disease outbreak news: avian influenza A(H5N1)—Cambodia; updated 8 February 2024 [cited 2024 Aug 5]. https://www.who.int/emergencies/disease-outbreak-news/item/2024-DON501

[13] World Health Organization. Disease outbreak news: avian influenza A(H5N1)—Australia; updated 7 June 2024 [cited 2024 Aug 12]. https://www.who.int/emergencies/disease-outbreak-news/item/2024-DON519

[14] Centers for Disease Control and Prevention. H5 bird flu: current situation [cited 2024 Aug 12]. https://www.cdc.gov/bird-flu/situation-summary/index.html

[15] Kons KM, Wood ML, Peck LC, Hershberger SM, Kunselman AR, Stetter C, et al. Exclusion of reproductive-aged women in COVID-19 vaccination and clinical trials. Womens Health Issues. 2022;32:557–63. 10.1016/j.whi.2022.06.004 36075817 PMC9197956

[16] Minchin J, Harris GH, Baumann S, Smith ER. Exclusion of pregnant people from emergency vaccine clinical trials: A systematic review of clinical trial protocols and reporting from 2009 to 2019. Vaccine. 2023;41:5159–81. 10.1016/j.vaccine.2023.06.073 37442686

[17] Centers for Disease Control and Prevention. Press release: highly pathogenic avian influenza A(H5N1) virus infection reported in a person in the U.S. [cited 2024 Aug 3]. https://www.cdc.gov/media/releases/2024/p0401-avian-flu.html

[18] Liu S, Sha J, Yu Z, Hu Y, Chan TC, Wang X, et al. Avian influenza virus in pregnancy. Rev Med Virol. 2016;26:268–84. 10.1002/rmv.1884 27187752

[19] Le TV, Phan LT, Ly KHK, Nguyen LT, Nguyen HT, Ho NTT, et al. Fatal avian influenza A(H5N1) infection in a 36-week pregnant woman survived by her newborn in Sóc Trăng Province, Vietnam, 2012. Influenza Other Respir Viruses. 2019;13:292–7. 10.1111/irv.12614 30291769 PMC6468084

[20] Shu Y, Yu H, Li D. Lethal avian influenza A (H5N1) infection in a pregnant woman in Anhui Province, China. N Engl J Med. 2006;354:1421–2. 10.1056/NEJMc053524 16571888

[21] Liu Y, Li Q, He YX, Zhang Y, Wen LY, Wang M, et al. [The firstly confirmed pregnant woman case of avian influenza A (H5N1) by etiological research in China] [in Chinese]. Bing Du Xue Bao. 2007;23:429–33. 18092678

[22] Shuang L, Yang C, Li Z, et al. Clinical analysis of the first maternal patient infected with novel avian influenza A(H5N6) virus in the world [in Chinese]. Zhonghua Wei Zhong Bing Ji Jiu Yi Xue. 2016;28:988–93.

[23] Wang G, Zhou Y, Gong S, Dong H, Wu G, Xiang X, et al. A pregnant woman with avian influenza A(H7N9) virus pneumonia and ARDS managed with extracorporeal membane oxygenation. Southeast Asian J Trop Med Public Health. 2015;46:444–8. 26521517

[24] Guo Q, Zhao D, Dong F, Liu S, Chen Y, Jin J, et al. Delivery of fetus death with misoprostol in a pregnant woman with H7N9 avian influenza A virus pneumonia and ARDS. Crit Care. 2014;18:589. 10.1186/s13054-014-0589-7 25672440 PMC4210470

[25] Qi X, Cui L, Xu K, Wu B, Tang F, Bao C, et al. Avian influenza A(H7N9) virus infection in pregnant woman, China, 2013. Emerg Infect Dis. 2014;20:333–4. 10.3201/eid2002.131109 24457138 PMC3901503

[26] Ding H, Xie L, Sun Z, Kao QJ, Huang RJ, Yang XH, et al. Epidemiologic characterization of 30 confirmed cases of human infection with avian influenza A(H7N9) virus in Hangzhou, China. BMC Infect Dis. 2014;14:175. 10.1186/1471-2334-14-175 24678603 PMC3977889

[27] Li Q, Lan Y, Xu CL, Liu Y, Wu TS, Wen LY, et al. [Study on a fatal pregnant woman died from by avian influenza (H5N1)] [in Chinese]. Zhonghua Liu Xing Bing Xue Za Zhi. 2006;27:288–92. 16875528

[28] Centers for Disease Control and Prevention. Technical update: summary analysis of genetic sequences of highly pathogenic avian influenza A(H5N1) viruses in Texas, United States of America, 2024 [cited 2024 Dec 9]. https://www.cdc.gov/bird-flu/spotlights/h5n1-analysis-texas.html

[29] Caserta LC, Frye EA, Butt SL, Laverack M, Nooruzzaman M, Covaleda LM, et al. Spillover of highly pathogenic avian influenza H5N1 virus to dairy cattle. Nature. 2024;634:669–76. 10.1038/s41586-024-07849-4 39053575 PMC11485258

[30] Food and Agriculture Organization of the United Nations, World Health Organization, World Organisation for Animal Health. Updated joint FAO/WHO/WOAH assessment of recent influenza A(H5N1) virus events in animals and people. 2024 Aug 14 [cited 2024 Dec 9]. https://www.who.int/publications/m/item/updated-joint-fao-who-woah-assessment-of-recent-influenza-a(h5n1)-virus-events-in-animals-and-people

[31] Centers for Disease Control and Prevention. Technical update: summary analysis of genetic sequences of highly pathogenic avian influenza A(H5N1) viruses in Texas [cited 2024 Aug 2]. https://www.cdc.gov/bird-flu/spotlights/h5n1-analysis-texas.html

[32] Furey C, Scher G, Ye N, Kercher L, DeBeauchamp J, Crumpton JC, et al. Development of a nucleoside-modified mRNA vaccine against clade 2.3.4.4b H5 highly pathogenic avian influenza virus. Nat Commun. 2024;15:4350. 10.1038/s41467-024-48555-z 38782954 PMC11116520

[33] European Commission for Emergency Preparedness and Response Authority. Commission secures access to 665,000 doses of zoonotic influenza vaccines [cited 2024 Jul 20]. https://health.ec.europa.eu/latest-updates/commission-secures-access-665000-doses-zoonotic-influenza-vaccines-2024-06-11_en

[34] THL National Institute of Health and Welfare. Infection and vaccination: avian influenza vaccine. Helsinki: The Institute; 2024.

[35] Rasmussen SA, Jamieson DJ, Bresee JS. Pandemic influenza and pregnant women. Emerg Infect Dis. 2008;14:95–100. 10.3201/eid1401.070667 18258087 PMC2600164

[36] Krubiner CB, Faden RR, Karron RA, Little MO, Lyerly AD, Abramson JS, et al.; PREVENT Working Group. Pregnant women & vaccines against emerging epidemic threats: Ethics guidance for preparedness, research, and response. Vaccine. 2021;39:85–120. 10.1016/j.vaccine.2019.01.011 31060949 PMC7735377

[37] Chomistek AK, Phiri K, Doherty MC, Calderbank JF, Chiuve SE, McIlroy BH, et al. Development and validation of ICD-10-CM-based algorithms for date of last menstrual period, pregnancy outcomes, and infant outcomes. Drug Saf. 2023;46:209–22. 10.1007/s40264-022-01261-5 36656445 PMC9981491

[38] Blehar MC, Spong C, Grady C, Goldkind SF, Sahin L, Clayton JA. Enrolling pregnant women: issues in clinical research. Womens Health Issues. 2013;23:e39–45. 10.1016/j.whi.2012.10.003 23312713 PMC3547525

[39] Cunnington M, Messenheimer J. Chapter 17: pregnancy registries: strengths, weaknesses, and bias interpretation of pregnancy registry data. In: Bradley RJ, Harris RA, Jenner P, editors. International review of neurobiology, 83. New York: Academic Press; 2008. p. 283–304. 10.1016/S0074-7742(08)00017-2 18929089

[40] Global Vaccine Data Network. Data dashboards [cited 2024 Jul 15]. https://www.globalvaccinedatanetwork.org/Data-Dashboards

[41] Black SB, Chandler RE, Edwards KM, Sturkenboom MCJM. Assessing vaccine safety during a pandemic: Recent experience and lessons learned for the future. Vaccine. 2023;41:3790–5. 10.1016/j.vaccine.2023.04.055 37198019 PMC10184950

[42] Klein NP. Rapid cycle analysis to monitor the safety of COVID-19 vaccines in near real-time within the Vaccine Safety Datalink: myocarditis and anaphylaxis. Presented at: Advisory Committee on Immunization Practices COVID-19 vaccines meeting; August 30, 2021; Atlanta, Georgia, USA.

[43] Zambrano LD, Ellington S, Strid P, Galang RR, Oduyebo T, Tong VT, et al.; CDC COVID-19 Response Pregnancy and Infant Linked Outcomes Team. Update: characteristics of symptomatic women of reproductive age with laboratory-confirmed SARS-CoV-2 infection by pregnancy status—Unites States, January 22–October 2, 2020. MMWR Morb Mortal Wkly Rep. 2020;69:1641–7. 10.15585/mmwr.mm6944e3 33151921 PMC7643892

[44] Kayem ND, Benson C, Aye CYL, Barker S, Tome M, Kennedy S, et al. Lassa fever in pregnancy: a systematic review and meta-analysis. Trans R Soc Trop Med Hyg. 2020;114:385–96. 10.1093/trstmh/traa011 32125412 PMC7197258

[45] Newsome K, Alverson CJ, Williams J, McIntyre AF, Fine AD, Wasserman C, et al. Outcomes of infants born to women with influenza A(H1N1)pdm09. Birth Defects Res. 2019;111:88–95. 10.1002/bdr2.1445 30623611 PMC6771262

[46] Food and Agriculture Organization of the United Nations, World Health Organization, World Organisation for Animal Health. Joint FAO/WHO/WOAH preliminary assessment of recent influenza A(H5N1) viruses 23 April 2024 [cited 2024 Jul 30]. https://cdn.who.int/media/docs/default-source/global-influenza-programme/2024_04_23_fao-woah-who_h5n1_assessment.pdf

[47] Rasmussen SA, Jamieson DJ, Macfarlane K, Cragan JD, Williams J, Henderson Z; Pandemic Influenza and Pregnancy Working Group. Pandemic influenza and pregnant women: summary of a meeting of experts. Am J Public Health. 2009;99(Suppl 2):S248–54. 10.2105/AJPH.2008.152900 19461110 PMC4504360

[48] Rasmussen SA, Jamieson DJ. Coronavirus disease 2019 and pregnancy is déjà vu all over again. BJOG. 2022;129:188–91. 10.1111/1471-0528.16859 34379870 PMC8441905

